# Incidence of Hematoma Following Breast Reduction in Patients Taking Selective Serotonin Reuptake Inhibitor or Serotonin-Norepinephrine Reuptake Inhibitor: A Retrospective Review

**DOI:** 10.7759/cureus.69323

**Published:** 2024-09-13

**Authors:** Anna Duncan, Krystal Stewart, Todd Dow, Jason Williams

**Affiliations:** 1 Division of Plastic and Reconstructive Surgery, Dalhousie University, Halifax, CAN; 2 Division of Plastic and Reconstructive Surgery, University of Manitoba, Winnipeg, CAN

**Keywords:** breast reduction, coagulation inhibitors, hematoma, selective serotonin reuptake inhibitor (ssri), serotonin-norepinephrine reuptake inhibitor (snri)

## Abstract

Background

Selective serotonin reuptake inhibitor (SSRI) and serotonin-norepinephrine reuptake inhibitor (SNRI) use is more common in the plastic surgery population compared to the general population. This study was designed to assess the theoretical effect of SSRIs and SNRIs on platelet function and the potential for increased bleeding risk. This study sought to establish the incidence of postoperative bleeding following routine bilateral breast reduction for patients on SSRIs or SNRIs. The outcomes of this study contribute to the discussion of whether these medications should be discontinued before elective surgery.

Methodology

A retrospective chart review of all patients who received bilateral breast reduction surgery over a 10-year period was performed. Patient charts were reviewed for postoperative hematoma formation as well as medications being used around the time of surgery. The rate of hematoma formation in patients actively taking SSRIs or SNRIs at the time of surgery was compared with the rest of the study population.

Results

A total of 1,022 patients met the inclusion criteria for the study. The overall incidence of postoperative hematoma was 7.7%. Of these, 1.9% of patients had clinically significant hematomas that required operative evacuation, and the remaining were treated conservatively. The only variable associated with a significantly higher risk of hematoma formation was advanced age (p = 0.005).

Conclusions

There was no significant difference in hematoma incidence after breast reduction in patients taking SNRIs or SSRIs compared with the general population. This contradicts some of the previously published literature and can hopefully guide clinicians in counseling their patients preoperatively.

## Introduction

Selective serotonin reuptake inhibitors (SSRIs) and serotonin-norepinephrine reuptake inhibitors (SNRIs) are widely prescribed antidepressant medications for the treatment of major depressive disorders and anxiety disorders [1]. Antidepressant use has increased by approximately 65% in the United States within the span of 15 years, from 1999 to 2014 [1]. Plastic surgery patients, particularly those undergoing breast surgery between the ages of 18-70 are more likely to be on psychoactive drugs compared to the general population [2].

The majority of serotonin within the human body is stored within platelets, and serotonin is known to enhance platelet aggregation through several mechanisms [3]. Lowering of intracellular serotonin levels within platelets through the use of SSRIs has been shown to reduce platelet function through inhibition of platelet plug formation and reducing platelet activation [3]. Hematomas are one of the most common acute complications of breast reduction surgery [4]. Hematomas typically present with significant pain, breast enlargement, and erythema. If large enough, prompt surgical evacuation and hemostasis are warranted to minimize the risk of further complications [4].

SSRI and SNRI use perioperatively has been associated with an increased risk of post-surgical bleeding events across surgical specialties, including a reported four-fold increased risk of hematoma in aesthetic breast procedures [5-7]. Despite this, no formal recommendation exists that SSRIs and SNRIs be discontinued before elective plastic surgery. There are appreciable concerns when considering cessation of these medications, including serotonin discontinuation syndrome and the risk of relapse of depression or anxiety symptoms [2]. The purpose of this retrospective review was to determine if there is any correlation between SSRI/SNRI use and post-surgical bleeding events after breast reduction surgery in comparison to non-SSRI/SNRI users.

## Materials and methods

A multi-centered, retrospective chart review was performed on all patients within the study province who received a bilateral breast reduction over a 10-year period (April 2008 to March 2018). Patients who were not receiving a routine bilateral breast reduction, patients who were taking other routine anticoagulation medication, or those who were lost to follow-up were excluded from the study. Patient demographics, medical history including active medications, operative details, and postoperative complications were collected from chart review. Postoperative bleeding events were noted, including clinically significant hematomas requiring operative evacuation as well as more minor hematomas treated conservatively with either observation or bedside aspiration.

Descriptive statistics were used to analyze postoperative hematoma formation. Categorical variables were expressed as frequency and percentage and continuous variables were expressed as mean ± standard deviation. Differences between categorical variables were assessed using the chi-square test or Fisher’s exact test. Differences between continuous variables were assessed using Student’s t-test. A full logistic regression model with all predictor variables was run for the formation of postoperative hematoma. All analyses were performed using SPSS Statistics for Windows, version 28.0.1.1 (14) (IBM Corp., Armonk, NY, USA), and a p-value of 0.05 or less was considered statistically significant.

## Results

**Figure 1:**
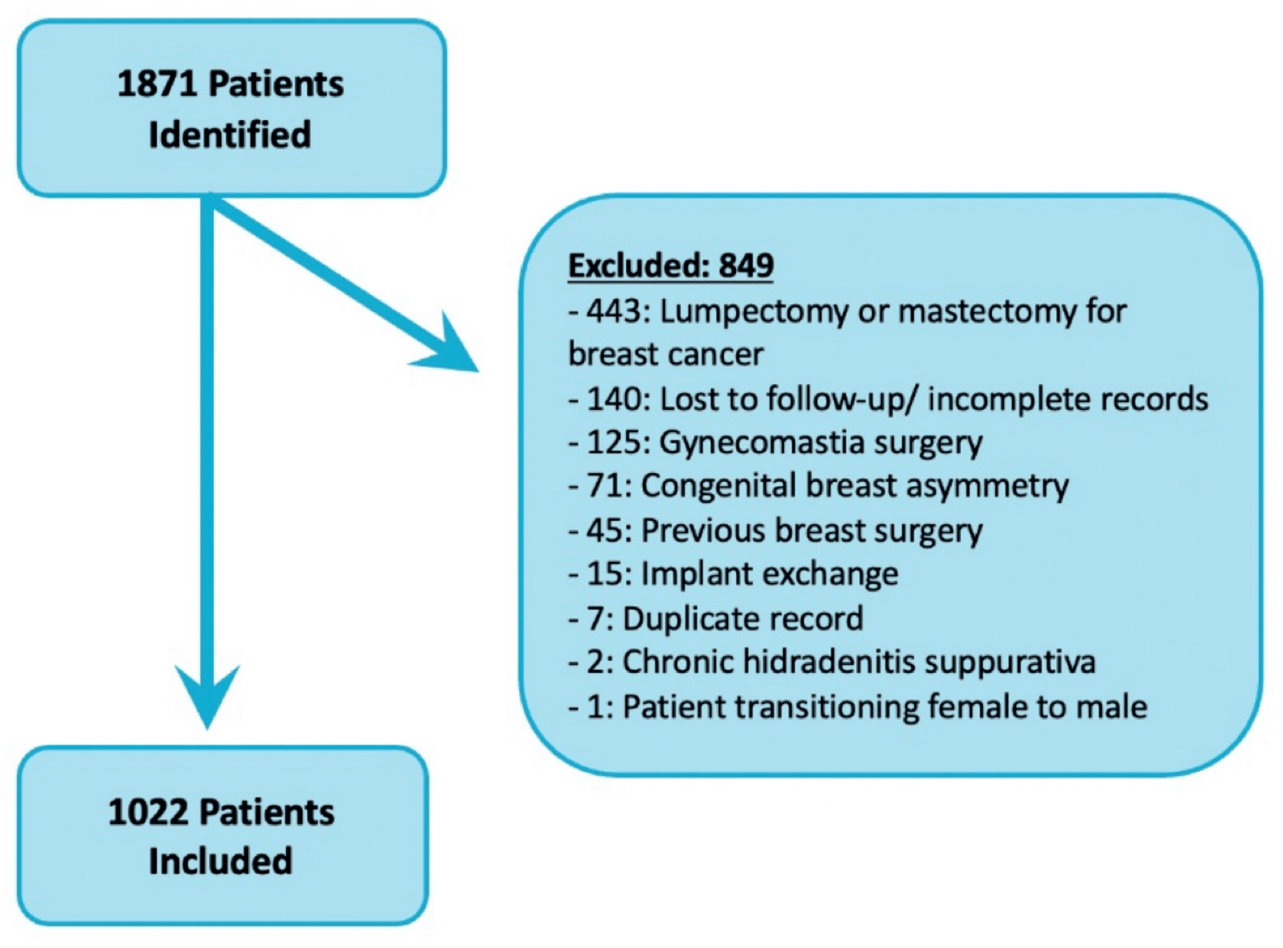
Inclusion criteria used to determine the patients included in the study.

Within the 10-year period of the study, 1,871 patients were identified by billing codes as having undergone breast reduction surgery. Once exclusion criteria were applied, 1,022 patients were included in the study (Figure 1). The mean age of the patient population was 43.4 ± 13.3 years (Table 1). The majority (54.9%) of the patients were treated at a teaching hospital and therefore likely had resident involvement in their care. Patients had an average of three postoperative follow-up assessments. A minority of the overall patient population was smoking at the time of surgery (8%). The most common medical comorbidity of the patient population was depression (23%), followed by gastroesophageal reflux disease (17%) and hypertension (14%).

**Table 1:** Patient variables of the study population and comparison with patients who developed postoperative hematomas. BMI: body mass index; SSRI: selective serotonin reuptake inhibitor; SNRI: serotonin-norepinephrine reuptake inhibitor

Variable	Overall patient population (n = 1,022)	Postoperative hematoma (n = 79)	No postoperative hematoma (n = 943)	P-value
Age (mean ± SD)	43.4 ± 13.3	47.4 ± 11.8	43.1 ± 13.4	0.005
BMI (mean ± SD)	25.9 ± 3.7	25.5 ± 3.5	25.9 ± 3.7	0.427
Smoker (n (%))	84 (8.2%)	8 (10.1%)	76 (8.1%)	0.539
SNRI use (n (%))	65 (6.4%)	5 (6.3%)	60 (6.4%)	0.964
SSRI use (n (%))	138 (13.5%)	12 (15.2%)	126 (13.4%)	0.669
Clotting disorder (n (%))	12 (1.2%)	3 (3.8%)	9 (1.0%)	0.059
Volume resected from each breast (mean ± SD)	619.2 ± 316.5	564.8 ± 330.8	623.7 ± 315.0	0.112
Wise reduction technique (n (%))	727 (71.1%)	61 (77.2%)	666 (70.7%)	0.233
Liposuction used (n (%))	221 (21.6%)	10 (12.7%)	211 (22.4%)	0.052

The most common preoperative symptom was back pain (87%), followed by neck pain (75%). Most patients were treated with a Wise-pattern reduction (71%). Furthermore, an inferior parenchymal breast pedicle was commonly utilized (68%). Liposuction was used in addition to surgical resection in 23% of patients. The overall incidence of a patient developing any complication postoperatively was 38% (n = 385), with the third most common complication being postoperative hematoma (8%).

Of the 79 patients noted to develop a postoperative hematoma, 37 (47%) underwent initial surgery and follow-up at a major tertiary center. Seventeen (46%) were managed clinically, with several requiring aspiration in the clinic, but the majority were managed conservatively. Overall, 20 patients with postoperative hematoma (1.95%) required immediate return to the operating theater within 24 hours of surgery for evacuation of the hematoma. Of the patients who developed hematomas with resident involvement in the initial surgery 44% occurred on the resident reduction side. Of the patients who required immediate return to the operating theater for hematoma evacuation, 50% occurred on the resident side, 40% on the staff surgeon side, and 10% occurred bilaterally.

When comparing the rate of hematoma formation postoperatively, the only patient variable that differed significantly was age (p = 0.005), with the patients developing hematomas having a slightly higher mean age (47.4 ± 11.8 compared to 43.1 ± 13.4 years). There was no significant difference in patients taking SNRI or SSRI and the incidence of hematoma formation postoperatively (Table 1). Multivariate logistic regression also demonstrated similar results, with age being a predicting variable for the development of a postoperative hematoma (p = 0.005). SSRI or SNRI use was not a predictive variable for the development of hematoma postoperatively. The predictive variables for hematoma formation are presented in Table 2.

**Table 2:** The results of the logistic regression model for postoperative hematoma formation. BMI: body mass index; SSRI: selective serotonin reuptake inhibitor; SNRI: serotonin-norepinephrine reuptake inhibitor

Variable	Odds ratio	Confidence interval	P-value
Age	1.027	1.008-1.047	0.005
BMI	0.974	0.906-1.047	0.472
Smoker	1.248	0.572-2.726	0.578
SNRI use	0.919	0.357-2.365	0.862
SSRI use	1.151	0.614-2.158	0.662
Clotting disorder	3.265	0.819-13.018	0.094
Mean volume resected from each breast	0.999	0.999-1.001	0.234
Wise reduction technique	0.930	0.482-1.791	0.827
Liposuction used	0.589	0.257-1.348	0.210

Of the 189 patients taking SSRI or SNRI medication, 65 patients were taking SNRI, with venlafaxine being the most common medication (n = 52), followed by duloxetine (n = 10). In our sample population, 138 patients were taking SSRIs, with citalopram being the most common (n = 44), followed by escitalopram (n = 35). Fourteen patients were taking two medications.

## Discussion

We hypothesized that the use of SSRIs would increase the risk of postoperative hematoma. The findings of our study, however, contradict our hypothesis. Perioperative use of SSRIs and SNRIs conferred no statistically significant increased risk of developing even minor postoperative hematomas.

Several other studies exist that have examined the heightened risk of postoperative bleeding associated with SSRI/SNRI use across surgical specialties, with an increased risk estimated to be as high as 36% [5,6,8]. These studies did not specifically look at breast reduction patients, and so the applicability to our study population is limited. In the breast surgery population, there have been prior reports of increased incidence of re-operation due to post-surgical bleeding in breast cancer patients actively taking SSRIs [9]. The most similar study to ours by Basile et al. looked at multiple elective breast procedures and SSRI-associated hematoma risk. They found a 4.14-fold increased risk of developing a hematoma which was indiscriminate of the procedure type [7].

We found that hematomas were the third most common postoperative complication within this study, affecting 8% of patients. Although this may seem high, it should be noted that we included all mentions of “hematoma” in the clinical chart, including those managed conservatively. Hematomas requiring operative evacuation were much lower at 1.95%, which is similar to the incidence reported by Basile et al. [7]. Interestingly, our study found that the only statistically significant risk factor for postoperative hematoma was increased age.

Due to the retrospective nature of this study, one major limitation was that there was no standardization of medication dosage or duration of treatment. Patients taking an SSRI or an SNRI varied in the type of medication prescribed, with some individuals on more than one medication. SSRIs were more commonly prescribed than SNRIs, with 73% of users on an SSRI. There was no means to determine medication compliance, and it is possible that some patients may not have been actively taking the medication at the time of surgery. Another limitation worth noting is that there may be variability among different surgeons in the documentation of perioperative medications and postoperative complications. Furthermore, we eliminated several patients due to loss to follow-up and/or incomplete records, which is a common limitation of retrospective studies.

Some factors could explain the significant difference between our findings and those previously reported [7]. In the aforementioned study, the proportion of the study population actively taking SSRIs was only 8.58% compared to 19.8% in our study population. It should be considered that with a relatively smaller sample size, validity may be compromised. This study also did not account for hematomas that required conservative treatment such as bedside aspiration, and so it is possible that this may have confounded the results.

Based on the findings of this study, we would not routinely recommend discontinuing SSRIs or SNRIs before breast reduction, as we were unable to detect any increased risk of hematoma formation. Furthermore, the benefits of these medications and the risk of abrupt discontinuation outweigh even the theoretically increased risk of bleeding. This study did have several limitations, mainly its retrospective nature, and further prospective research is required to definitively assess the risk of bleeding events associated with SSRI/SNRI use in plastic surgery patients. This is especially pertinent given that many plastic surgery procedures are elective and can be delayed to mitigate the risk of postoperative complications.

## Conclusions

This study investigated the risk of post-breast reduction hematoma in SSRI/SNRI users and found that there was no significant difference compared with the general population. Currently, there is no routine recommendation to discontinue SSRIs/SNRIs before elective breast reduction surgery and our findings are in accordance with this. There is conflicting prior research, but ultimately the risk of discontinuation outweighs the benefits, especially given that no prospective studies have demonstrated an increased hematoma risk. We would advise clinicians to counsel their patients accordingly on hematoma risk, but not to advise discontinuing psychoactive medications to mitigate this risk based on the literature available.
